# What Accounts for Physical and Emotional Health? Influence of Health, Personality, and Health Activation

**DOI:** 10.1093/geroni/igaa057.740

**Published:** 2020-12-16

**Authors:** Joseph Kim, Nicholas Cone, Rotem Arieli, Peter Martin

**Affiliations:** Iowa State University, Ames, Iowa, United States

## Abstract

The purpose of this study was to identify associations among health personality, health activation, and emotional and physical health, and to identify direct and indirect effects. Participants in the study consisted of 3907 older adults, 65 years of age and older. Measures used in the analyses were the Health Personality Assessment, the Consumer Health Activation Index, and The Veterans RAND 12-Item Health Survey. Structural equation modeling with bootstrap sampling estimation was conducted to examine direct and indirect effects. The measurement model, X2(307)=2142.34, CFI=0.96, RMSEA=0.04, and structural model, X2(313)=2167.36, CFI=0.96, RMSEA=0.04 yielded an acceptable fit. Significant direct paths were observed between health personality factors and health activation, and in turn health activation to emotional and physical health. The results indicate that older adults with lower levels of Health Neuroticism, lower Health Openness, higher Health Agreeableness, and higher Health Conscientiousness had higher levels of health activation. In addition, older adults with higher levels of health activation had higher emotional and physical health. Also, direct paths from health personality to emotional and physical health were observed. Lastly, significant indirect effects were health activation had a significant positive indirect effect on physical health through Health Agreeableness. Health activation had a significant negative indirect effect on emotional health through Health Neuroticism and Health Openness. The implication of this study is that health activation has a significant role in the emotional and physical health of older adults through health personality dispositions. In addition, health personality factors directly influence the emotional and physical health of older adults.

